# The impact of the social environment on Zambian cervical cancer prevention practices

**DOI:** 10.1186/s12885-018-5164-1

**Published:** 2018-12-12

**Authors:** Anayawa Nyambe, Jarl K. Kampen, Stridutt K. Baboo, Guido Van Hal

**Affiliations:** 1 University of Antwerp, Antwerp, Belgium; 2 Wageningen University, Wageningen, Netherlands; 3 University of Zambia, Lusaka, Zambia

**Keywords:** Zambia, Cervical Cancer, Screening, Vaccination, Social ecological model, Theory of triadic influence

## Abstract

**Background:**

Cervical cancer which is preventable by screening and vaccination is the most common cancer in Zambia among both the female and male population. In this article we aim to determine how the key players of the sociocultural and political environment recognize cervical cancer as a public health problem and therefore impact the provision of cervical cancer prevention services (screening and vaccination).

**Methods:**

Qualitative data in the form of interviews with stakeholders (health care providers, teachers and religious leaders), special interest groups (advocacy groups and non-governmental organizations) and policy makers, was collected as part of a mixed methods study from February to May 2016.

**Results:**

The views expressed by the respondents were coded into predetermined themes (cervical cancer in general, screening, vaccination) and an organizational chart of the administration of cervical cancer prevention services in Zambia was developed.

**Conclusions:**

It is evident that the Zambian cervical cancer prevention system has targeted several areas and multiple sectors of society to reduce cervical cancer cases. However, awareness, knowledge, social support and facilities are factors that can be improved.

**Electronic supplementary material:**

The online version of this article (10.1186/s12885-018-5164-1) contains supplementary material, which is available to authorized users.

## Background

Most women worldwide are at risk of cervical cancer which is relatively easy to prevent by both primary (vaccination) and secondary (screening) prevention methods. To prevent the spread of cervical cancer in Zambia, the Cervical Cancer Prevention Program (CCPPZ) was started in 2006 [2, 14]. Under this program, nurses were trained to provide free cervical screening via visual inspection with acetic acid (VIA) and treatment with cryotherapy if indicated [14, 17]. Women with complex cervical cancer lesions are referred to the University Teaching Hospital (UTH) for further evaluation [14, 16, 17]. Peer educators are employed to facilitate community awareness, counteract misconceptions and myths, and provide patient support functions [4, 14]. In addition to this, screening services are also provided by [21], Project Concern International (PCI) for mobile screening [13] and in chiefdoms for village-based screening approaches [20]. Currently, cervical screening has been made available in all 10 provinces of Zambia [12].

During 2013, Zambia launched the Human papillomavirus (HPV) vaccine Gardasil as a demonstration [26]. This pilot program primarily targeted girls in grade 4 using a school-based vaccination strategy which also included girls aged 10 years old who were not in schools [11]. Male circumcision is also being encouraged because the cancer is sexually transmitted and it reduces HPV infection in men by 30% [23].

Regardless of these initiatives, cervical cancer is estimated to be diagnosed in 2330 Zambian women every year and 1380 die from the disease [1]. Unfortunately, the true size of the problem is unknown because of under reporting and lack of reliable data [2, 27]. Nevertheless, the Zambian cervical cancer prevention program may be more effective if support from stakeholders (professional groups, policy makers) and adequate infrastructure (equipment, institutions, capital) are considered.

### Purpose of this study

Existing studies of populations in Zambia are very few and are limited because they only focus on women’s screening practices [10, 22, 25] and views on vaccination [8]. These studies have not considered how the environment that women reside, facilitates the provision of these prevention services. The purpose of this study is to determine how the key players of the sociocultural and political environment recognize cervical cancer as a public health problem and therefore impact the provision of cervical screening and vaccination services. The data is used to sketch an organizational chart on the administration of cervical cancer prevention services in Zambia.

The Social Ecological Model (SEM) and the Theory of Triadic Influence (TTI) are the theoretical frameworks that guided this study. These models were selected because they cover multiple ecological levels of influence, share similar concepts and can be used to assess screening and vaccination health behaviors. The SEM is composed of the intrapersonal, interpersonal, organizational, community, and policy levels [9]. The TTI comprises of the ultimate, distal, and proximal levels of influence as well as intrapersonal, interpersonal social, and sociocultural environmental streams of influence [6]. The Environmental level of the TTI is comparable to a combination of the organizational, community and policy levels of the SEM. For a systematic review on the SEM and TTI about how these models have been applied in health behavior studies and recommendations for their usage in screening and vaccination research, see [15]. The SEM and TTI suggest factors that predict/influence health behavior, in this case screening and vaccination practices, which were used to develop study instrument topics based on the three predetermined themes (cervical cancer in general, screening, and vaccination). (The term *general* refers to anything parting to cervical cancer which is not related to screening and vaccination services; *screening* refers to any activities in relation to cervical screening; *vaccination* refers to any activity in relation to vaccination of cervical cancer). The predetermined themes were selected because this research focuses on screening, vaccination and all other aspects of cervical cancer. The factors of health behaviour as applied in this study are in Table 1. In accordance to these frameworks, the implementation and availability of cervical cancer services to the general public was assessed by considering the contribution of health care providers, teachers and pastors; advocacy groups and nongovernmental organizations (NGOs); (The terms *advocacy groups* and *nongovernmental organizations* are used to refer to any organization or group that is not affiliated to the government of Zambia); and policy makers.

**Table 1:** Factors of the SEM and TTI modified and applied in this study

*SEM* ^a^	*TTI* ^b^ *Environment level* (Stakeholders, Special interest groups, Policy makers)	Factors that influence behaviour as applied in the interview guides of the current study
*Organizational* (Stakeholders) - Social institutions with organizational characteristics, and formal/informal rules and regulations for operation.	*Ultimate* Aspects of women’s immediate surroundings, neighbourhoods, social institutions, and culture that, although beyond the personal control of women, put them at risk	• availability of services^c^ • employment rates^c^ • inadequate schools^c^ • media depictions^c^ • (worksite) policies that vaccination and screening^d^
*Community* (Special interest groups) - Relationships among organisations, institutions and informal networks within defined boundaries.	*Distal* Values and behaviours of women that contribute to their attitudes toward screening and vaccination	• information opportunities (media and advertisements) • interaction with social institutions (conducting campaigns) • knowledge^c^ • stigma, values and evaluations (religious beliefs, cultural norms, barriers/uptake, priority, prevalence)
*Public policy* (Policy makers) - Local, state, and national laws and policies	*Proximal* Beliefs and evaluations about the benefits of cervical cancer prevention	• age recommendations of screening and vaccination • possibility of self-screening and cost • possibility of vaccinating girls and boys

^a^SEM definitions of levels from [3]; [9]

^b^TTI definitions of levels from [19]

^c^Only the TTI mentions this factor

^d^The factor “policy”, is mentioned in the SEM at both organizational and public policy level

## Methods

### Study design, site and population

A cross-sectional mixed methods study was carried out from February to May 2016. In this article, emphasis is only on qualitative data obtained by means of interviews because of the large amount of data collected (by focus group discussion and quantitative questionnaires). The qualitative interview data was collected as a more effective way to dialogue with the respondents on the phenomena of human health behaviour regarding cervical cancer and its prevention. In accordance with a qualitative phenomenological approach [18], interviews were conducted with respondents who are familiar with cervical cancer to gain an in-depth understanding. The design involved triangulation of data sources by collecting information from different groups of people as well as theory triangulation [24], by applying both the SEM and TTI (see Table 1) instead of only a single model to improve validity of results.

The criterion required for respondents to participate in this research was that they were located in Lusaka City and be at least 18 years old. Based on the SEM and TTI, the respondents included stakeholders (health care providers, teachers, pastors), special interest groups (NGOs, advocacy groups), and policy makers. Media and educational administration offices were included to fill-in gaps on the functioning and organization of the cervical cancer prevention program. It was decided to focus on Chilenje and Kanyama for information from stakeholders at clinics, schools, and churches. These areas were chosen because of the availability of cervical screening services at the local clinic, the population density, and the difference in living standards between the two areas with Chilenje being more developed than Kanyama. The groups that were targeted are summarized in Table 2.

**Table 2:** Summary of target groups

Group	Location	Target^a^	Method
Stakeholders	Chilenje, Kanyama & other areas in Lusaka	Health care providers (UTH, CDH, Kanyama and Chilenje Clinics)	Interviews (*N* = 17)
	Chilenje & Kanyama	Head teachers (schools)	Interviews (*N* = 13)
	Chilenje & Kanyama	Religious leaders (churches)	Interviews (*N* = 7)
Special Interest groups	Other areas in Lusaka	Non-governmental organizations, advocacy groups (CIDRZ/ACEWCC, DDMU, INESOR, MUVI TV, PCI, PPAZ, Q TV/Radio, THPAZ, WHO, Zambia Daily Mail, ZICTA, ZNBC)	Interviews (*N* = 20)
Policy makers	Other areas in Lusaka	Government officials (DEB, MCDMCH, MoH)	Interviews (*N* = 4)

^a^Abbreviations in order of appearance in the table: University Teaching Hospital (UTH), Cancer Disease Hospital (CDH), Centre for Infectious Disease Research in Zambia (CIDRZ), African Centre of Excellence for Women’s Cancer Control (ACEWCC), District Disaster Management Unit (DDMU), Institute of Economic and Social Research (INESOR), Project Concern International (PCI), Planned Parenthood Association of Zambia (PPAZ), Traditional Health Practitioners Association of Zambia (THAPZ), World Health Organization (WHO), Zambia Information and Communications Technology Authority (ZICTA), Zambia National Broadcasting Association (ZNBC), District Education Board (DEB), Ministry of Community Development Maternal and Child Health (MCDMCH), Ministry of Health (MoH)

### Study instruments and development

The authors of this research developed semistructured interview guides based on the health behavior factors of the SEM and TTI as mentioned in Table 1 in the background. Each factor mentioned by the models was operationalized into a research question, the questions were either available from previous literature or developed by the authors. The topics included information about the institution, cervical cancer in general, effectiveness of services provided, school-based vaccination program, information sources, and finally views on cervical screening and vaccination. These topics then formed the basis for three predetermined themes of cervical cancer in general, screening, and vaccination used for analysis. Additional file 1 has the interview guides [see Additional file 1].

### Sampling design

Sampling was conducted in two steps, first selecting the target institution and then selection of study participants. Judgmental sampling methods were used to select institutions for participation in the research so that information is from knowledgeable individuals. This was facilitated by reviewing literature to determine which institutions have been involved in cervical cancer prevention. Meeting with these institutions also provided further information on other target institutions. Once an institution was selected, in person visits were made to describe the study and schedule interviews. Interviews at clinics and hospitals were conducted by random selection of health care providers in permitted departments once permission was obtained from authorities. The random selection was done conveniently by approaching everyone who was available and interviewing those who were willing to participate. At schools, churches, NGOs/advocacy groups, media, and government offices, interviews were conducted with key informants based on referral from the institutional authorities. One-on-one semistructured interviews lasting approximately 30 min on average were conducted at the workplace of the respondent for convenience and safety. If a respondent refused to have their interview voice recorded it was written. Effort was made to ensure that the written reports were as accurate as possible, and questions were repeated to verify the written responses. All interviews were conducted in person except for one interview guide that was left as a drop-off form.

### Data analysis

All interview data was compiled in the computer program NVivo. Some interviews were voice recorded and transcribed. Interviews that were not voice recorded (*N* = 35) were because of requests made by respondents. Guided by the topics of the interviews, the data was read and coded into three predetermined themes (cervical cancer in general, screening, and vaccination). The transcribed data, written interviews and notes were reviewed by identifying the key words and phrases under the theme. The key phrases and words that fit together were grouped into emerging categories. These categories were then refined and reduced by grouping similar categories together. Findings were then reported based on these categories. In addition, the reported role that a respondent/department played in the cervical cancer prevention was used to create the organizational chart of the administration of cervical cancer prevention services in Zambia.

## Results

**Fig. 1:**
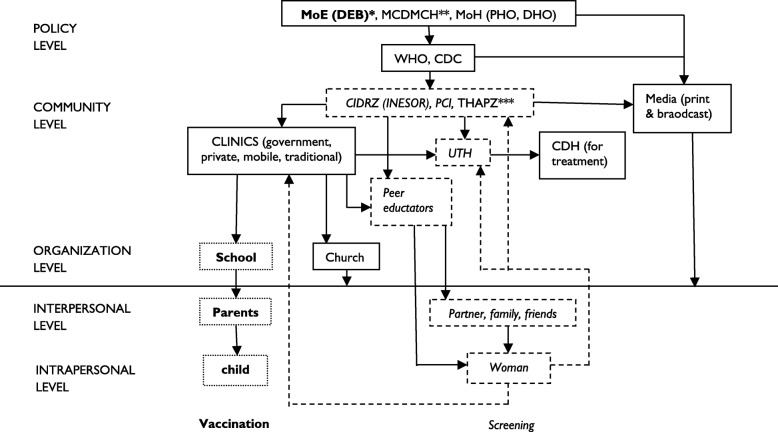
Organizational chart of the cervical cancer prevention process in Zambia (according to departments visited). Abbreviations in order of appearance: Ministry of Education (MoE), District Education Board (DEB), Ministry of Community Development Maternal and Child Health (MCDMCH), Ministry of Health (MoH), Provincial Health Office (PHO), District Health Office (DHO), World Health Organization (WHO), Centers for Disease Control and Prevention (CDC), Center for Infectious Disease Research in Zambia (CIDRZ), Institute of Economic and Social Research (INESOR), Project Concern International (PCI), Traditional Health Practitioners Association of Zambia (THAPZ), University Teaching Hospital (UTH), Cancer Disease Hospital (CDH). Regular text facilitated both screening and vaccination; Bold text-dotted box only facilitated vaccination; Italic text-dashed box only facilitated screening. *MoE were key partners in vaccination only. ** MCDMCH has currently been changed to the Ministry of Community Development and Social Welfare and the departments of maternal and child health are back under the MoH. ***THPAZ does not provide screening or vaccination services but traditional health practitioners might refer possible cervical cancer cases to health care providers

A total of *N* = 61, interviews were conducted. The respondents were above legal age of 18 years old and resided in Lusaka City. The reported involvement of the respondents created the organizational chart (Fig. 1). The three predetermined themes are used to present the results of the analysis as reported by policymakers, special interest groups (advocacy groups, NGOs, media) and stakeholders (health care providers, teachers, church leaders).

### Administration of cervical cancer prevention services in Zambia

Figure 1 illustrates the organizational chart of the administration of cervical cancer prevention services in Zambia with consideration given to the social and ecological levels of influence (intrapersonal, interpersonal, organizational, community and policy level). This figure was developed based on the reported involvement of respondents and their institutions in providing cervical cancer prevention services in Zambia. An additional file has an explanation on the components of Fig. 1 and how each item interacts [see Additional file 2].

### Cervical cancer in general

#### Importance of cervical cancer

According to policy makers and special interest groups, cervical cancer is an important health issue in Zambia as evident from statistics. Special interest person 1 said, “[Looking at] the number of people with HIV since the two diseases go together, [cervical cancer] is a big problem. [It is] not as common as malaria but the outcome is normally death.” Similarly, some stakeholders said there are other health problems of equal importance to cervical cancer as illustrated by quote 1 in box 1 in Additional file 2 [see Additional file 2]. For health care providers, this view depended on their department: “I think cervical cancer is not given enough attention [considering] on average about 5 cases of cervical cancer [are detected] everyday by biopsy. When you are away from [cervical cancer] you don’t see it.” (Health care provider 1).

#### Worksite policy

Policymakers reported that there is no policy concerning cervical cancer prevention only a strategic plan which was being finalized. Only teachers and health care providers at clinics said there is a policy for the provision of the vaccine. Clinics were actively involved in both screening and vaccination compared to hospitals that were more involved in screening.

#### Workforce

Apart from a few health care providers who said that the number of staff in their department is enough for vaccination provision, the majority of the respondents (policymakers, special interest groups and stakeholders) said that the workforce for provision of screening and vaccination services was limited and overworked (quote 2 & 3 box 1, Additional file 2). Policy makers further reported that lack of finances makes it difficult to employ more nurses. They suggested that having a way of informing the community when nurses work outside the clinic (e.g. in schools, training programs) might improve the management system. Traditional healers suggested that collaborations are needed since they cannot provide blood transfusions, drip, oxygen or operations and equally doctors cannot provide exorcisms, treat demons and ghosts (quote 4, box 1, Additional file 2).

#### Information sources

Policy makers stipulated that they inform the public on cervical cancer though the media, meetings at schools for vaccine awareness, the mHealth project which will use SMS text messages and through existing community structures (traditional marriage counselors) (quote 5, box 1, Additional file 2). Special interest groups further included running community outreach programs, social media sites (Facebook), using brochures and brand ambassadors. Churches reported having health talks, health departments and encouraging members seek health care (quote 6, box 1, Additional file 2). Whether health care providers provided information on cervical cancer depended on their department or their patient’s signs (quote 7, box 1, Additional file 2). Teachers had meetings with health care providers and were given materials (handbooks, posters) for talking to pupils and parents though some had a different experience (quote 8, box 1, Additional file 2). “The girls were not given leaflets about the HPV vaccine. [The nurses] only left a poster to stick in the class and we wrote memos to the parents about the vaccine,” said teacher 1.

The respondents (policy makers, special interest groups, stakeholders) believe that the general public gains information by word-of-mouth (quote 9, box 1, Additional file 2). Policy makers also mentioned informal meetings (kitchen parties, church gatherings) and social media, while stakeholders mentioned peer educators, media, brochures, posters, workshops and public announcements (in churches, schools, markets).

#### Effectiveness of sensitization

Special interest groups and stakeholders agreed that more was needed for sensitization (quote 10, 11, & 12, box 1, Additional file 2):

The media is not doing enough. People should go into the community and sensitize the country. People don’t (.. .) screen unless they have symptoms. In fact, even among nurses (.. .) there is not enough sensitization. [Nurses] don’t know the cost of screening, (.. .) if it’s free or if doctors recommend [it]. (Health care provider 1).

Respondents from special interest groups suggested that brochures should be printed in local languages and not only English to maximize coverage. The media (television, radio) was generally considered effective. The media reported having health related programs and some networks also had a health desk devoted to coving health related topics. They requested that health experts should have a more permanent partnership with the media by making themselves available when needed, assisting in program sponsorship and building capacity in health care reporters. Stakeholders recommended sensitizing the community by having workshops, hospital consultations and using public figures (quote 13, box 1, Additional file 2). Health care provider 2 said, “The former first lady [used to be] on television all the time talking about cervical cancer and we had an overwhelming response. The [patients] who were coming told us that [they] heard about it from the first lady.”

Policy makers thought that Zambians living in urban areas like Lusaka are more informed about cervical cancer than those in rural areas. They suggested that providing excellent services at clinics might improve practice.

#### Knowledge

During discussions with the public, special interest groups reported talking about symptoms (bleeding), causes (HPV, traditional practices including douching and intravaginal insertion of herbs in particular tobacco), prevention (male circumcision, washing of foreskin, condoms, behavior change, screening, monogamy), and treatment (temporal break from sexual activity, clinical treatment, herbal remedies for early stages) (quote 14, box 1, Additional file 2). Traditional healers included more causes of cervical cancer as mentioned by special interest person 2, “causes I mention[ed are] witchcraft, (.. .) unhygienic conditions, (.. .) bad sexual habits, bad methods of sex, and sometimes drugs. Herbal drugs which a woman inserts [in her vagina] that cause friction during [intercourse] and bruises the cervix.” Some churches had unique views on cervical cancer causes (diet i.e. meat eating, spiritual attack, allowed by God), prevention (by having the spirit of God, faith) and treatment (by faith, prayers, divine healing, trusting the will of God, casting out spirits of sickness, using anointed items).

#### Social support

Special interest groups reported that men were interested in cervical cancer prevention and offering support to women (quote 15, box 1, Additional file 2). Health care providers had a contrary view (quote 16, box 1, Additional file 2):

There are [a] few [women] that are supported by [their] husband. The few that I have seen are those that have post-coital bleeding. The husband gets concerned and escorts his wife to find out why she is bleeding after sex all of a sudden. Otherwise most women walk in by themselves. (Health care provider 2).

“Most men are uncircumcised and the uncircumcised carry the virus. Men should be involved but not everyone is willing to be circumcised though most are doing so now,” said health care provider 3.

### Screening

#### Screening age recommendation

Unlike health care providers, the stakeholders at churches and schools had no involvement in the screening program. Policy makers specified that screening age will be from 25 to 59 years old. This is based on the natural history of the disease, country level data over the past few years and HIV/AIDS prevalence:

HIV infected [women] should start screening as soon as they become sexually active. [There is] no age range or screening interval [for them]. For HIV unknown or negative screening starts at 25. Previously we used to say, ‘As long as you are sexually active.’ (Policy maker 1).

In spite of these guidelines, special interest groups and health care providers were not fully aware on the screening age recommendations. Some health care providers specified that screening should be started from puberty (10, 12 or 15 years old) because of the difficulty of certifying virginity or from reproductive ages. Most respondents from special interest groups and health care providers said screening should be conducted as long as a woman was sexually active or from age 25 to 55 and above (quote 17 & 18, box 2, Additional file 2). Special interest group respondents further said that screening should be done every year regardless of HIV status, though it used to be every 2 or 3 years for HIV negative women.

#### Screening uptake and barriers

Policy makers stated that screening coverage in Lusaka was low and fear of dying drives screening uptake. The respondents (policy makers, special interest groups and stakeholders) generally agreed that screening uptake was facilitated by having awareness and knowledge. Policy makers and health care providers also said that knowing someone who had cervical cancer and partners support increases uptake of screening. Special interest groups and health care providers said women usually come for screening if they were curious about their health and if they have symptoms:

Zambians don’t go for health care if nothing hurts. (.. .) So people who have themselves checked are usually those who feel pain or something. [Then] because of sensitization and people learning about the cancer they can’t feel, they [decide] to get checked. That was the case for me. I was like, ‘Oh my gosh! You can have it and not feel it? Let me go check it.’ (Special interest person 3).

Apart from having limited screening facilities/programs (quote 19, box 2, Additional file 2), lack of awareness, lack of knowledge and fear (of dying, pain, unknown, stigma, a positive result) was sighted by most respondents (policy makers, special interest groups and stakeholders) as barriers to uptake of screening. Policy makers further said that partner’s influence in decision making (e.g. husband might refuse to abstain during treatment) as a barrier (quote 20, box 2, Additional file 2). Special interest groups and health care providers also added lack of symptoms and beliefs (cultural, religious, misconceptions) as barriers. Respondents from special interest groups further mentioned feeling shy of male doctors, procrastination and refusal to return for call-back as reasons of low uptake (quote 21, box 2, Additional file 2).

#### Self-sampled screening

According to policy makers and a few special interest groups, low resource HPV deoxyribonucleic acid (DNA) testing and self-sampling kits are being considered for introduction if they pass future feasibility trials. However, most respondents from special interest groups and health care providers had never heard about self-sampled screening and their views were varied. Their main concern was the ease of use (quote 22 & 23, box 2, Additional file 2).

### Vaccination

Unlike policy makers, District Health Office respondents, WHO respondents, healthcare providers at clinics and most teachers, respondents from other target sectors stated that they did not have any involvement in the pilot of the HPV vaccine and therefore did not have much information. Furthermore, vaccination for cervical cancer is beyond the scope of traditional healers although there are traditional ways of vaccination against measles and other diseases using herbs, tattoos, steaming, and/or talismans. All churches interviewed agreed that vaccination must be encouraged.

#### Vaccine administration

Policy makers reported learning several lessons in administration, vaccine handing and cost cutting by running the two vaccination demonstrations (quote 24, box 3, Additional file 2). They found that public schools were more likely to participate than private schools (quote 25, box 3, Additional file 2), and stakeholders noticed that not all schools participated in the vaccination program (quote 26 & 27, box 3, Additional file 2). Policy makers further noted that school based vaccination had a higher coverage and cost compared to facility based with the biggest challenge being accessing out of school girls:

Health seeking behavior in Zambia is not the best. [A woman] will not go to the clinic unless she is sick. (.. .) So expecting healthy people to voluntarily come for vaccination is difficult. [Furthermore], people are [protective of] their children and won’t voluntarily bring them. So we said it’s better we follow them. (Policy maker 2).

#### Vaccination age recommendation

Respondents from special interest groups and stakeholders were aware that the vaccine was administered to grade 4 girls and out of school girls age 10. Nevertheless, some schools were strict on vaccinating only 9 or 10 year old grade 4 girls (quote 28, box 3, Additional file 2). Health care providers at the hospital who gave their opinion on age had mixed views, ranging from only vaccinating virgins to giving adults the option (quote 29, box 3, Additional file 2): “It is better to be administered as a choice at adult ages,” said health care provider 1.

#### Vaccination uptake and barriers

It was reported by policy makers and stakeholders that uptake of the vaccine was due to having awareness, knowledge, fear of having or dying from cancer and knowing someone who had cancer. Policy makers also mentioned that well travelled people were more likely to request the vaccine. Stakeholders noticed that people who saw others practice vaccination without experiencing side effects made them want to have the vaccine (quote 30 & 31, box 3, Additional file 2). Policy makers believe that uptake of the vaccine will increase over time when people start seeking the vaccine themselves because of seeing the benefits.

Policy makers said sensitization on vaccination was effective but there was poor social mobilization because of low funds which lead to misunderstandings (age limit, side effects). Policy makers and stakeholders agreed that lack of awareness and knowledge was a barrier to vaccination uptake. Special interest groups and stakeholders further added fear of side effects (myth of infertility). Policy makers and stakeholders also said that religious beliefs (quote 32 & 33, box 3, Additional file 2) and policy restrictions (on age, gender) reduced uptake (quote 34, note 3, Additional file 2): “Even parents with children in older grades wanted the vaccine. Others were even tried to reduce their [age] to 10 years old. They were very much willing.” (Teacher 2).

#### Vaccination coverage

According to policy makers, vaccination coverage was higher in Zambia compared to other countries. Special interest groups commended the work of the MoH in sensitization that resulted to a relatively good vaccination uptake. Therefore, there are plans to do a national rollout as reported by policy maker 2, “When we do a national rollout because of the lessons that were learnt and scien[tific evidence]. We will not be using the 3 dose [vaccine. This] (.. .) will reduce cost. We will be doing the 2 dose [vaccine].” Limited resources make it difficult to remove restrictions on age range as well as gender even though special interest groups and some stakeholders supported the vaccination of boys (quote 35, 36 & 37, box 3, Additional file 2). Teachers in particular disapproved the vaccination of boys because of nurses’ instruction (quote 38, box 3, Additional file 2), “[Parents] were encouraged to take boys for circumcision [and] most of them did it during the holiday that year. [Some boys] came to report that they had been circumcised. I gave them books and other materials for motivation,” said teacher 2.

## Discussion

Based on the results, the main factors influencing cervical cancer prevention practices in Zambia include policy, availability or lack of facilities and services, information sources and knowledge, as indicated by the SEM and TTI in Table 1. Knowledge is affected by religious beliefs that may not support some medical practices, cultural practices like intravaginal insertion of tobacco, and stigma expressed as fear e.g. cancer means death. Other factors that were found were social interactions which are at the interpersonal levels of the SEM and TTI [6, 9, 15].

### Policy

The more involved a person was in cervical cancer and its prevention, the more likely they believe that cervical cancer is a main health concern in Lusaka. However, during the time of the research, there was no policy governing cervical cancer prevention and the MoH was finalizing the strategic plan for improving Zambian health care. The lack of a functional strategic plan or policy has affected service provision and limited the information that is available to stakeholders. For instance, there were obvious inconsistencies in screening age recommendations. However, the vaccine was administered in an elaborate pilot program and most respondents generally accepted that vaccination was limited to grade 4 and 10-year-old girls as policy (MCDMCH, n.d.). It should be noted that some respondents had interest in vaccinating other ages and this could increase coverage. The effectiveness of vaccine program in comparison to the inconsistences of administration of screening services, supports the evidence that organized health programs facilitate cervical prevention services [7].

### Facilities and services

Most respondents stated that there was also a lack of adequate facilities and workforce for provision cervical cancer services. The vaccine, was at the time of this research administered as a pilot and some eligible schools did not participate in the vaccination program possibly because of poor registration of schools. Perhaps more efforts should be put into involving volunteers (youth from the community), using existing structures (traditional marriage counselors) and introducing other methods that might reduce the interval of screening (low cost HPV DNA test) or the need for personnel (self-screening kits). The introduction of self-sampled screening is being considered by the government and some NGOs, but there is need for sensitization because of lack of awareness. It is suggested to use provider directed self-sampled screening along with VIA, to supplement the existing equipment, solve problems of literacy, reduce fears of pain and give providers contact with women who might have other health problems.

### Information sources and knowledge

Although it was reported that the public is sensitized on cervical cancer, some nurses, teachers and church leaders do not even practice prevention or know where services are provided. Lack of awareness, fear and misconceptions greatly limited uptake of prevention practices as identified by previous studies [4, 8, 10]. This has probably reason it was reported women seek screening services in late stages of cervical cancer development when symptoms are present. It was also reported that parents were hesitant to vaccinate their daughters because of fear of future sterility and religious beliefs. Clearly, there is a need for more information sharing among stakeholders who have contact with the public to ensure that incorrect facts, religious and cultural beliefs that might increase the risk of cervical cancer are not encouraged.

### Social interactions

As evident from the results, very few men support their partners during cervical treatments. It is recommended to include men in cervical cancer campaigns because they affect decision making and influence the behavior of women [5, 7]. Furthermore, since vaccination of boys will not be considered at a national level, more emphasis should be given to circumcising boys. Then at an appropriate age, boys should be made aware of cervical cancer, taught condom use and remaining faithful to their partners when in relationships.

### Limitations and outlook

The limitations include location of the study which was limited to Lusaka which is not representative of the whole of Zambia. The views of the churches, schools and clinics might have been different if selected from other areas within Lusaka. Sampling bias could have occurred by using judgmental and volunteer sampling methods. Some of the targeted participants were not willing or available to participate. Regardless of this, these were suitable sampling methods as they allowed easy access to respondents who were interested in contributing their views in a short period of time. The triangulation of data also helped to increase confidence in the data collected. Once the strategic plan is functional, a follow-up study is recommended to see if views and practices have changed.

## Conclusions

The Zambian cervical cancer prevention system has targeted several areas and multiple sectors of society to reduce cervical cancer cases. The SEM and TTI were effective in selection of study groups and determining factors that influence health seeking behavior. The effectiveness of these models is evident in the illustration of the organizational chart of cervical cancer prevention services in Zambia, that can be arranged according to these frameworks most especially the SEM. However, the application of these models in this study was limited because social interactions which were identified during interviews when respondents mentioned their interactions with women and men were not targeted. Furthermore, certain study groups were not able to comment on screening and/or vaccination because they were not actively involved in service provision. This was most especially noticed on the topic of self-screening, where lack of information made it difficult for respondents to give their opinion on cost and acceptability.

The research question was what is the impact of the sociocultural and political environment on facilitating and hindering the practice of cervical screening and vaccination in Zambia? The sociocultural and political environments form the basis of the cervical cancer prevention program and they play an integral role in facilitating and supporting women in the uptake of screening. They make it possible for parents to allow their daughters to be vaccinated against HPV. However, this is limited by having insufficient staff, facilities and the lack of policy. The general low health seeking behavior among Zambians is a hindrance to cervical cancer prevention. In spite of all sectors agreeing that cervical cancer is an important health issue, being a silent infection makes it difficult to fight.

## Additional files

Additional file 1: Interview Guides. The questions used to guide interviews. (DOCX 43 kb)

Additional file 2: Supporting Results Data. Administration of cervical cancer prevention services in Zambia and boxes containing quotes from respondents. (DOCX 46 kb)

## Abbreviations

ACEWCC: African Centre of Excellence for Women’s Cancer Control
AIDS: Acquired Immune Deficiency Syndrome
CCPPZ: Cervical Cancer Prevention Program in Zambia
CDC: Centers for Disease Control and Prevention
CDH: Cancer Disease Hospital
CIDRZ: Centre for Infectious Disease Research in Zambia
DDMU: District Disaster Management Unit
DEB: District Education Board
DHO: District Health Office
DNA: Deoxyribonucleic acid
ECZ: Examinations Council of Zambia
HIV: Human Immunodeficiency Virus
HPV: Human Papilloma Virus
INESOR: Institute of Economic and Social Research
MCDMCH: Ministry of Community Development Maternal and Child Health
MoE: Ministry of Education
MoH: Ministry of Health
nd: not dated
NGO: Nongovernmental organization
PCI: Project Concern International
PHO: Provincial Health Office
PPAZ: Planned Parenthood Association of Zambia
SEM: Social Ecological Model
SMS: Short Message Service
THPAZ: Traditional Health Practitioners Association of Zambia
TTI: Theory of Triadic Influence
UTH: University Teaching Hospital
VIA: Visual Inspection with Acetic Acid
WHO: World Health Organization
ZDF: Zambia Defense Force
ZICTA: Zambia Information and Communications Technology Authority
ZNBC: Zambia National Broadcasting Corporation
