# Supplementary material for: The impact of the social environment on Zambian cervical cancer prevention practices
Source: BMC Cancer. 2018 Dec 12;18:1242. doi: 10.1186/s12885-018-5164-1 (PMC6292082; doi:10.1186/s12885-018-5164-1)
Supplement: Supplementary file 1 — Interview Guides. The questions used to guide interviews. (DOCX 43 kb) [file 12885_2018_5164_MOESM1_ESM.docx]

**INTERVIEW FOR STAKEHOLDERS (healthcare providers, head teachers, pastors)**

Date:

Interview round #:

Name of institution:

Occupation of participant:

Your institution:

1. a) Is this a private, public, religious or non-religious school/hospital?

b) What Christian denomination is this church?

Cervical cancer:

1. a) How familiar are you with cervical cancer?

b) How common is cervical cancer in Lusaka?

1. How familiar are you with cervical cancer screening? What is your experience?
2. How familiar are you with the vaccine to prevent cervical cancer? What is your experience?

Effectiveness of service:

1. What do you know about the availability of cervical screening and vaccination services in Lusaka? What resources (such as education, services, counseling, etc.) are you familiar with, if any?
2. a) In your opinion, do you think the number of doctors/nurses at your clinic is enough to cater to the population?

b) In your opinion, do you think the number of churches, schools are enough to help facilitate and educate the public on cervical cancer prevention?

1. Are there any worksite policies/regulations/program that require you to:

Educate the public on cervical screening and vaccination

Participate in administering vaccination and screening services

Why/why not?

School based vaccination program:

1. a) Did this school participate in the school based cervical cancer vaccination program? Why or why not?

b) Do you think the number of schools in this area was adequate enough to administer the school based vaccine? Why or why not?

c) In spite of the vaccination program being school based, did anyone else other than school girls request the vaccine including older women and men? What were the characteristics of those people?

Information and sources:

1. Do you recommend screening and vaccination to your patients/ congregation/ parents and students?
2. Do you feel that the current media depictions of cervical cancer prevention are effective in sensitizing the public? Why or why not?
3. What promotional and outreach strategies might increase screening and vaccination? Can you think of any creative strategies you’ve used that have worked well?

Views:

1. What factors facilitate decisions to seek screening and vaccination?
2. What barriers to seeking screening and vaccination exist? Can you think of a strategy you’ve used to overcome these barriers?
3. What age range do you recommend for screening? Why?
4. What are your views on self-screening? What are views on payment for self-screening?
5. What are your views on vaccinating girls as well as boys?
6. What age range would you recommend for cervical cancer vaccination?

Finally:

1. Do you consider cervical cancer to be one of the main health concerns in Lusaka? Why or why not?
2. Do you have any further information you would like to share, or any documents that you feel may be essential for this study?

**INTERVIEW FOR SPECIAL INTEREST GROUPS**

Date:

Interview round #:

Name of institution:

Occupation of participant:

Cervical cancer:

1. a) How familiar are you with cervical cancer?

b) How common is cervical cancer in Lusaka?

1. How familiar are you with cervical cancer screening? What is your experience?
2. How familiar are you with the vaccine to prevent cervical cancer? What is your experience?

Effectiveness of service:

1. What do you know about the availability of cervical screening and vaccination services in Lusaka? What resources (such as education, services, counseling, etc.) are you familiar with, if any?
2. In your opinion, is the workforce adequate enough to sensitize the public on cervical cancer prevention? If not, where is it lacking?
3. Are there any worksite policies/regulations/programs that require you to:

Educate the public on cervical screening and vaccination

Administer vaccination and screening services

Why/why not?

School vaccination program:

1. Do you feel that the school based vaccination program adequately covered Lusaka? Why or why not?
2. In spite of the vaccination program being school based, did anyone else other than school girls request the vaccine including older women and men? What were the characteristics of those people?

Information and sources:

1. In your opinion, where do people in Lusaka generally get information about cervical cancer (church, school, clinics, media)?
2. Do you feel that the current media depictions of cervical cancer prevention are effective in sensitizing the public? Why or why not?
3. What promotional and outreach strategies might increase screening and vaccination? Can you think of any creative strategies you’ve used that have worked well?

Views:

1. What factors facilitate decisions to seek screening and vaccination?
2. What barriers to seeking screening and vaccination exist? Can you think of a strategy you’ve used to overcome these barriers?
3. What age range do you recommend for screening? Why?
4. What are your views on self-screening? What are views on payment for self-screening?
5. What are your views on vaccinating girls as well as boys?
6. What age range would you recommend for cervical cancer vaccination?

Finally:

1. Do you consider cervical cancer to be one of the main health concerns in Lusaka? Why or why not?
2. Do you have any further information you would like to share, or any documents that you feel may be essential for this study?

**INTERVIEW FOR POLICYMAKERS**

Date:

Interview round #:

Name of institution:

Occupation of participant:

Cervical cancer:

1. a) How familiar are you with cervical cancer?

b) How common is cervical cancer in Lusaka?

1. How familiar are you with cervical cancer screening? What is your experience?
2. How familiar are you with the vaccine to prevent cervical cancer? What is your experience?

Effectiveness of service:

1. In your opinion, is the workforce i.e. healthcare providers, educators (teachers, pastors), advocates in healthcare, adequate enough to sensitize the public on cervical cancer prevention? If not, where is it lacking?
2. Basically, what are the current policies in place for administering screening and cervical cancer vaccination (priority population, regulations)? How do you feel these policies could be made more effective?
3. What factors should be considered when recommending policies for cervical cancer vaccination and cervical screening among risk population groups?

School based vaccination program:

1. Do you feel that the school based vaccination program adequately covered Lusaka? Why or why not?
2. In spite of the vaccination program being school based, did anyone else other than school girls request the vaccine including older women and men? What were the characteristics of those people?

Information and sources:

1. In your opinion, where do people in Lusaka generally get information about cervical cancer (church, school, clinics, media)?
2. Do you feel that the current media depictions of cervical cancer prevention are effective in sensitizing the public? Why or why not?
3. What promotional and outreach strategies might increase screening and vaccination? Can you think of any creative strategies you’ve used that have worked well?
4. What do you know about the availability of cervical screening and vaccination services in Lusaka? What resources (such as education, services, counseling, etc.) are you familiar with, if any?

Views:

1. What factors facilitate decisions to seek screening and vaccination?
2. What barriers to seeking screening and vaccination exist? Can you think of a strategy you’ve used to overcome these barriers?
3. What age range do you recommend for screening? Why?
4. What are your views on self-screening and the possibility of introducing it to the Zambian population? What are views on payment for self-screening?
5. What are your views on vaccinating girls as well as boys?
6. What age range would you recommend for cervical cancer vaccination?

Finally:

1. Do you consider cervical cancer to be one of the main health concerns in Lusaka? Why or why not?
2. Do you have any further information you would like to share, or any documents that you feel may be essential for this study?
