# Supplementary material for: The impact of the social environment on Zambian cervical cancer prevention practices
Source: BMC Cancer. 2018 Dec 12;18:1242. doi: 10.1186/s12885-018-5164-1 (PMC6292082; doi:10.1186/s12885-018-5164-1)
Supplement: Supplementary file 2 — Supporting Results Data. Administration of cervical cancer prevention services in Zambia and boxes containing quotes from respondents. (DOCX 46 kb) [file 12885_2018_5164_MOESM2_ESM.docx]

**Supporting Results Data**

**Administration of cervical cancer prevention services in Zambia as Reported by Respondents**

*Policy Level*

It was reported that the Ministry of Health (MoH) played the main role in organizing and implementing both screening and vaccination prevention programs. Collaborations were made with the Ministry of Education and the Ministry of Community Development Maternal and Child Health Child Unit for the vaccination program. Collaborations were also made with the departments of Mother Health, Clinical Care and diagnostic services and Mobile and Emergency Health Services for cervical screening. The District Health Office implements policies made by the MoH. The Lusaka Urban District Health Management Team operates the public health clinics in Lusaka and the University Teaching Hospital (UTH). The District Disaster Management Unit is only called if the MoH fails to contain a disease. Furthermore, developmental partners and United Nations agencies like World Health Organization primarily offered technical support. The Centers for Disease Control and Prevention through the United States President's Emergency Plan for Acquired Immune Deficiency Syndrome (AIDS) Relief was the major funder of this program.

*Community Level (composed of Non-Governmental Organizations [NGOs], health care facilities and media)*

It was reported that the Centre for Infectious Disease Research in Zambia (CIDRZ) has an e-hub department (for training health care providers, consultation), community department (for peer educators), data unit (for data analysts, call back system), and clinics. CIDRZ has played a major role by working with the Institute of Economic and Social Research to conduct cervical cancer research, collaborating with the MoH in cervical screening skills training of nurses, development of screening services at government clinics, provision of mobile clinics and the sensitization of the public by use of peer educators. Peer educators work under clinics or NGOs to educate the public on cervical cancer and its prevention.

Project Concern International works with the Zambia Defense Force though mobile clinic programs for the provision of free screening using Visual Inspection with Acetic Acid, HIV/AIDS testing and sensitization of the public. The Traditional Health Practitioners Association of Zambia is the main organization in Zambia that unites traditional healers. The association works to ensure that traditional health practitioners deliver primary health care for women only up to early stages of cervical cancer infection and emphasizes the need of referral of their patients to clinics. Planned Parenthood Association of Zambia (PPAZ) has four family planning clinics in Zambia with nurses that provide fee paying screening services via pap smear. PPAZ also conducts community outreach programs to sensitize the public. The media through print (Zambia Daily Mail), television and radio (Zambia National Broadcasting Cooperation, QTV/radio, MUVI) is involved in sensitizing the public by either their own efforts or from the prompting of the policy makers and/or NGOs.

Government clinics provide screening and vaccination services. MoH in collaboration with CIDRZ started a cervical cancer screening program with 65 clinics nationwide that were chosen based on location, population, infrastructure (space, human resource). At clinics patients can only receive the diagnosis of a suspected cancer, if needed patients are then referred to the UTH for further analysis and the diagnosis of cervical cancer. Patients in provinces have their specimen sent for analysis at UTH. Once definitive diagnosis is made the patients are referred to the gynecology department or the Cancer Disease Hospital for treatment depending on the staging.

*Organizational Level*

It was reported that the vaccination program was conducted at schools. Churches might assist in sensitization of the public.

*Interpersonal and Intrapersonal Level*

It was reported that the main influence of behavior was parents that consent to have their children vaccinated. Additionally, partners, family and friends who heard about screening from churches, health care providers and the media influence women’s decision to practice screening.

**Result Boxes**

| **Box 1: Cervical Cancer General** |
| --- |
| *Importance of cervical cancer*   1. “No [cervical cancer] is not the main health problem. There is malaria, HIV, and [currently] cholera outbreaks.” (Teacher 1)   *Workforce*   1. “When I'm on leave it is closed. So therefore anyone who comes will be told to wait [un]til I come back or go to other clinics within Lusaka that provide screening.” (Health care provider 2) 2. “The challenge was not many health personnel. They only considered big schools and they would tell those in smaller schools to bring the pupils to the big school for vaccination. It would have been better to follow them there.” (Teacher 2) 3. “If I know the cancer is in early stages and manageable then the prescription and treatment is my own. (…) A second opinion is recommended for advanced cases because we do not have equipment. So we refer to people with equipment, people who can examine, people who can verify and give us findings. Cervical cancer is claiming a lot of lives. If we had a three way referral mechanism: healer-to-healer, healer-to-clinic and maybe clinic-to-healer, we could be saving a lot of lives.” (Special interest person 2)   *Information sources*   1. “We tried especially [in the] rural setting [with] traditional marriage councilors who basically give information about marriage, (. . .) child bearing, [and] pregnancy, generally they talk about reproductive health and to the point, (. . .) was to increase their knowledge on cervical cancer. [They would then use this] to communicate to a community which is not openly talking about such things because the [community] knows this is a group that is legalized to talk about such things.” (Policy maker1) 2. “Two nurses at this church talk about health issues during the main service. They give talks once or twice a year usually on cholera. The nurses came and asked for permission and I as the pastor gave them the go ahead. I felt it would be good for the congregation. They do it only twice because people know it all.”(Church leader 1) 3. “Screening is recommended to those who have symptoms or are found to have a cervix that is not fine. Those who have a complication are referred.” (Health care provider 4) 4. “There was no explanation. The nurses came and told us to arrange our pupils for vaccination. Then we sent consent forms to parents. The parents that signed and agreed got their daughters vaccinated.” (Teacher 3) 5. “We found that (. . .) the highest form of getting information is from a friend (. . .) like a family friend, somebody you talk to [e.g. if] I go for screening and tell somebody else about it or a mother goes and encourages the daughters to [screen].” (Policy maker 1)   *Effectiveness of sensitization*   1. “I think much has to be done. Look at the way HIV is being talked about, information is available almost everywhere. But we have clients who say they have never heard about cervical cancer [and the] client lives in Lusaka.” (Special interest person 4) 2. “The number of churches and schools are not enough to help facilitate and educate the public. The community has many pupils who do not go to school and may not even go to church. Therefore, a door to door campaign would do.” (Teacher 4) 3. “The hindrance is the culture. Especially as a priest to talk to the congregation about the cancer is culturally difficult to mention in church. As a priest I can say or talk about women’s health but not cervical cancer.” (Church leader 2) 4. “Some places are youth friendly. The youth can be used to sensitize schools and they will hear from fellow youth which would be easy to take.” (Health care provider 4)   *Knowledge*   1. “Apart from douching a lot of women like inserting herbs. At the moment the most common herb is ‘*nsukno*’ (tobacco). They drink it to make their bodies very warm. They insert it inside their vagina to make it very tight, warm [and] dry. But men tell us, ‘we don’t tell [women] to do that it’s on their own’. Women say, ‘our men want dry sex and our vaginas to be very tight’.” (Special interest person 4)   *Social support*   1. “[Men are told that] it is important to use condoms. But married men are very difficult (. . .), they say this is my wife and I cannot use a condom. So we tell them to be faithful to one another. (. . .) If you are married stick to your partner but if you are not married and you have multiple partners please try to use condoms.” (Special interest person 4) 2. “Most of the women who come [to the cancer ward] have already been left by their husbands before coming here. So three quarters of the [women] who are here were once married.” (Health care provider 5) |

| **Box 2: Cervical Cancer Screening** |
| --- |
| *Screening age recommendation*   1. “I remember enrolling one client who was 15 years old because children of nowadays start having sex at a very tender age. For us, as long as you have slept with a man (. . .) then you have to come for screening. That’s what we normally tell them.” (Special interest person 4) 2. “The age is 25 and above. It’s up to 59 I think for our case. For me personally I would even screen a 70 year old if she has never been screened. I would screen because compared to advanced countries (. . .) we are a low resource setting. People have never had screening and they may only have a chance when they are 70.” (Health care provider 2)   *Screening uptake and barriers*   1. “We have about 17 [screening instrument] sets. So once we see (. . .) 17 clients it means we are done for the day. (. . .) We tell clients who arrive later to come the following day (. . .) because we have limited instruments that cannot be reused. Sometimes they take time to come again. Some clients will give excuse[s], ‘I started menstruating so I couldn’t come’ or, ‘yesterday I had this program’ but after some time they do come.” (Special interest person 4) 2. “We have still not reached screening target in Lusaka. Underserved women are most likely sick. Men and other factors affect decision making.” (Policy maker 1) 3. “At times our patients complain about us calling them just to remind them about screening. They say, ‘why are you calling me? Is it a satanic movement or what? (. . .) I went to church and the pastor prayed for me so I am OK I don't need your services anymore’.” (Special interest person 5)   *Self-sampled screening*   1. “Though it is a good idea I don’t think it would be necessary at this time because most of the women that are screened are (. . .) illiterate. So (. . .) levels of education need to use that kind of test kit must be considered.” (Special interest person 5) 2. “I don’t think it would work for Zambian people. Maybe rich people, high class but for low and the middle class since it takes 15 minutes to screen at the clinic, I don’t know if anyone would like to trade that and buy [a kit]. I wouldn’t do it. I rather just go every 3 years and have myself checked.” (Special interest person 3) |

| **Box 3: Cervical Cancer Vaccination** |
| --- |
| *Vaccine administration*   1. “We have done two demonstrations. The first demonstration was done from 2013 to 2014 with completed 3 doses given. Overall coverage of the fully immunized was 58%. A second demonstration was done [with consideration given to] the lessons [learned] (. . .) to see how it will work at the national [scale]. We also remained with enough vaccines to do a second demonstration and we didn’t want to waste them.” (Policy maker 2) 2. “Those are policy decisions that are going to be made. You have countries like South Africa which has basically decided they are not funding the private schools (. . .). The government has focused on government schools.” (Policy maker 1) 3. “We had a series of meetings with the school but they refused to the point they told us ‘Go vaccinate somewhere else not our school’. Some of the parents of children at that school brought their daughters straight to the clinic for the vaccine.” (Health care provider 4) 4. “Everyone (schools) just had to participate. Once the ministry sends out the circular, then the clinic made a time-table to say when they will be coming to that school to administer the vaccine.” (Teacher 5)   *Vaccination age recommendation*   1. “For the [entire] grade 4. Every one of them even if they are 16 years old they are vaccinated because they are in grade 4. The age of 16 is a bit rare but a 15 year old in grade 4 is more common and that is in government schools. In private schools mostly 9 to10 years old. Government school children are older. [The nurses] just follow the policy but at 16 years old, they screen them by asking, ‘if they have ever met a man’ to see if they are virgins. They say they have not. If they have there is no use to vaccinate them. Age depends on how a person has kept herself.” (Health care provider 4) 2. “It should not be a choice but it should be mandatory to negate the negative beliefs or myths e.g. ‘I can’t get the virus so why should I be vaccinated?’ So it’s better mandatory.” (Health care provider 1)   *Vaccination uptake and barriers*   1. “The ones that agreed their parents were very willing. They were happy to say the government developed the program. Others were saying they lost their relative to the same disease so they agreed. One parent was undergoing chemotherapy that time who also decided to support the daughter.” (Teacher 2) 2. “No side effects reported only the usual, swelling of the infected area, pain, redness.” (Teacher 2) 3. “About maybe two complained that they were sick about five parents said that their kids will be infertile. The girls who got sick after where the ones who came on an empty stomach. They were told to eat.” (Teacher 6) 4. “[Some] grade 4 pupils started the vaccine but never finished the doses. Some transferred [and for] others their parents [didn’t allow] them to take the second dose. Then some refused completely because they feared the girls would be sterile in the future, ‘Zambia has a large population so you are trying to reduce the number’. Also, ‘They are just considering our children because we are poor in the compound and you are just trying to experiment on our children’. To combat the beliefs, we continued talking to them. One case (. . .) the father refused but the daughter wanted it. So we followed the daughter’s request but had to stop anyway. Others were religious beliefs, ‘We are Christians and we only believe in prayer and anointing water that’s all’.” (Teacher 2) 5. “Others wanted the vaccine but the policy blocked them. Some parents complained by saying, ‘My grade 4 child is 10 years old and my grade 7 child is also 10 years old but only the grade 4 child is vaccinated, why not both?’“ (Health care provider 4)   *Vaccination coverage*   1. “Parents have asked about vaccinating boys and they have been told to take their male children for circumcision.” (Health care provider 4) 2. “It was targeting girls only because they have the cervix. Men have the virus and therefore it is better to vaccinate the women because she gets the disease. It is her to suffer.” (Health care provider 6) 3. “It would be a good idea to vaccinate boys as well because there are boys who are abused by older women in their homes.” (Teacher 2) 4. “No parents with boys wanted the vaccine because of the guidelines we were given. They were told boys infect girls so prevent the one who can get the HPV.” (Teacher 5) |
